# Defining the dynamic chromatin landscape of mouse nephron progenitors

**DOI:** 10.1242/bio.042754

**Published:** 2019-05-07

**Authors:** Sylvia Hilliard, Renfang Song, Hongbing Liu, Chao-hui Chen, Yuwen Li, Melody Baddoo, Erik Flemington, Alanna Wanek, Jay Kolls, Zubaida Saifudeen, Samir S. El-Dahr

**Affiliations:** 1 Department of Pediatrics, Section of Pediatric Nephrology, Tulane University School of Medicine, New Orleans, LA 70112, USA; 2 Department of Pathology & Tulane Cancer Center, Tulane University School of Medicine, New Orleans, LA 70112, USA; 3 Departments of Pediatrics & Medicine, Center for Translational Research in Infection and Inflammation, Tulane University School of Medicine, New Orleans, LA 70112, USA

**Keywords:** ATAC-seq, ChIP-seq, Epigenetics, Kidney, Nephrogenesis

## Abstract

Six2^+^ cap mesenchyme cells, also called nephron progenitor cells (NPC), are precursors of all epithelial cell types of the nephron, the filtering unit of the kidney. Current evidence indicates that perinatal ‘old’ NPC have a greater tendency to exit the progenitor niche and differentiate into nascent nephrons than their embryonic ‘young’ counterpart. Understanding the underpinnings of NPC development may offer insights to rejuvenate old NPC and expand the progenitor pool. Here, we compared the chromatin landscape of young and old NPC and found common features reflecting their shared lineage but also intrinsic differences in chromatin accessibility and enhancer landscape supporting the view that old NPC are epigenetically poised for differentiation. Annotation of open chromatin regions and active enhancers uncovered the transcription factor Bach2 as a potential link between the pro-renewal MAPK/AP1 and pro-differentiation Six2/b-catenin pathways that might be of critical importance in regulation of NPC fate. Our data provide the first glimpse of the dynamic chromatin landscape of NPC and serve as a platform for future studies of the impact of genetic or environmental perturbations on the epigenome of NPC.

## Abstract (precis)

**Summary:** An investigation of the chromatin landscape of mouse nephron progenitors across their life span supports the view that old nephron progenitors are epigenetically poised for differentiation.

## INTRODUCTION

Reciprocal interactions between the ureteric bud and surrounding nephron progenitor cells (NPC) of the cap mesenchyme govern nephron induction. The cap mesenchyme is composed of an early progenitor Cited1^+^/Six2^+^ compartment and a transit Cited1^−^/Six2^+^ compartment that subsequently differentiate into the pre-tubular aggregate, the precursor of the renal vesicle, the earliest epithelial precursor of the nephron (Brown et al., 2013; Mugford et al., 2009). Careful morphometric studies and cell cycle analyses have shown that the proportion of NPC progressing through the cell cycle decreases with NPC aging, whereas the contribution of cell death is minimal, suggesting that all NPC exit occurs via differentiation into early nephrons (Short et al., 2014). Using genetic and primary cell culture models, Fgf9 and Bmp7 were shown to stimulate the MAPK pathway activation of Fos and Jun in the cap mesenchyme leading to the formation of the AP-1 heterodimer which stimulates cell cycle and growth factor genes contributing to the maintenance of the NPC population (Muthukrishnan et al., 2015). Although Fgf9 levels do not fall appreciably during NPC development, Fgf20 levels do (Barak et al., 2012). It is therefore possible that reduced growth factor availability/activity in the niche is partly responsible for the short lifespan of NPC. However, there are also intrinsic differences between young and old NPC. For example, Six2 (and other stemness factors such as Wt1, Osr1 and Sall1) levels decline in postnatal NPC, suggesting that these low levels cannot sustain NPC stemness in the face of elevated canonical Wnt signaling. A decline in the glycolytic capacity has also been shown by RNA-seq on postnatal NPC (Chen et al., 2015) as well as in primary young and old NPC (Liu et al., 2017). These changes translate into differences in cell behavior as demonstrated in the heterochronic transplantation studies (Chen et al., 2015); whereas young NPC tend to remain in the progenitor niche, old NPC exit and differentiate. The biological underpinnings of NPC development, i.e. the greater tendency of perinatal NPC to differentiate compared to their embryonic counterpart, are not well understood.

Here, we compared the chromatin landscape of young and old NPC and found that dynamic chromatin accessibility to developmental enhancers is an intrinsic property of maturing NPC. Genome-wide ATAC-seq and ChIP-seq uncovered common and differentially accessible chromatin regions in young versus old NPC, reflecting their shared identity but also their maturational differences. Relative gain and loss of enhancer accessibility correlated with NPC gene expression and identified the poised epigenetic state of differentiation genes. While the open chromatin of young NPC is enriched in binding sites for the core NPC transcription factors (Six2, Wt1, Hoxa/c/d, Tead, AP1), old NPC gain chromatin accessibility to the Bach2/Batf complex, a repressor of AP1-mediated transcriptional activation. In summary, our data support the notion that dynamic changes in the NPC epigenome over their lifespan balance NPC proliferation and differentiation.

## RESULTS

### Identification of cell types in mouse Six2 nephron progenitors

**Fig. 1.**
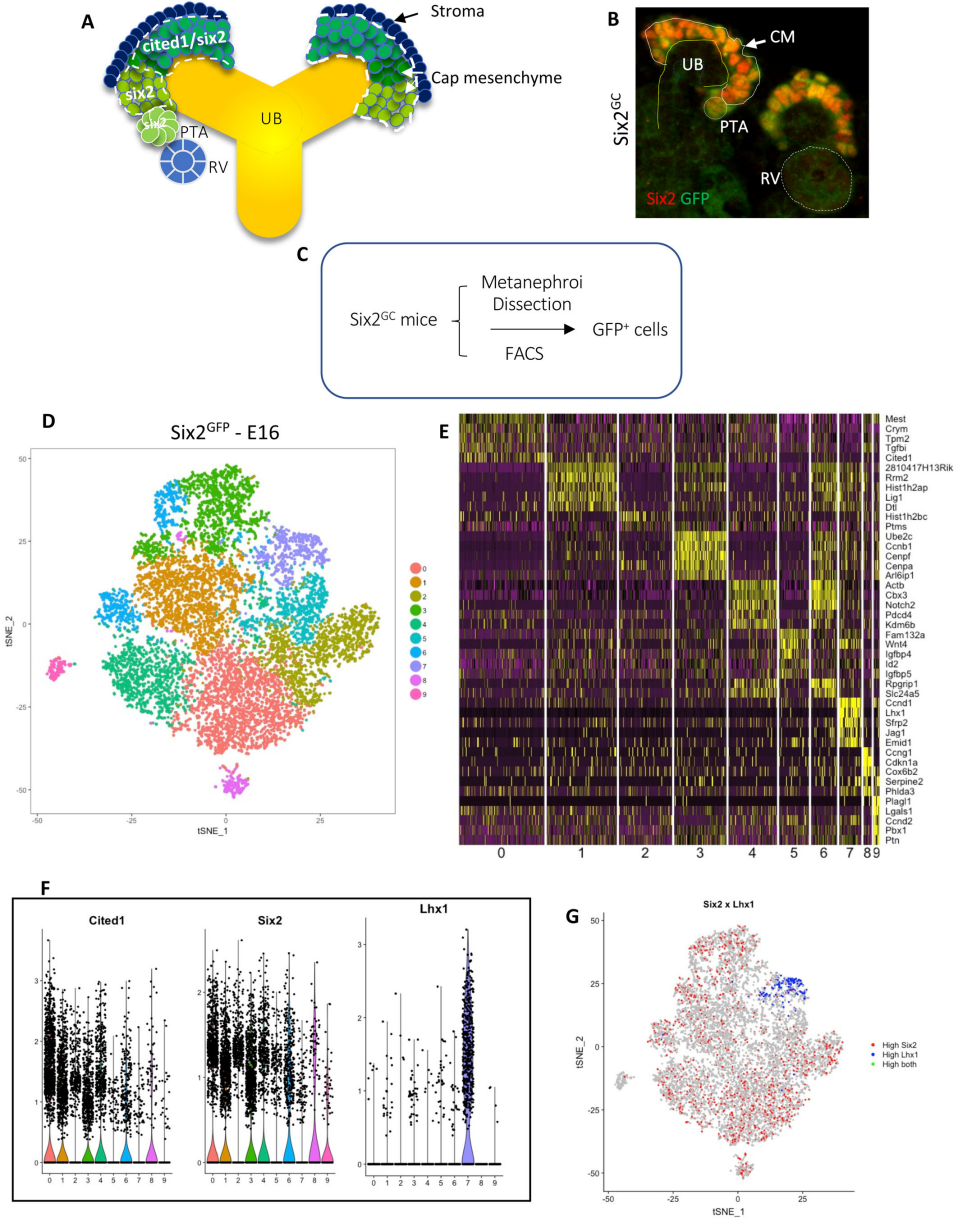
**Assessment of mouse nephron progenitor cell (NPC) diversity.** (A) Schematic of the nephrogenic niche and compartments of the cap mesenchyme based on expression of Cited1 and Six2. (B) Kidney tissue section from E16.5 Six2^GC^ mouse co-stained with Six2 and GFP antibodies. High levels of Six2/GFP are present in the cap mesenchyme whereas low levels are observed in the pre-tubular aggregate. UB, ureteric bud; CM, cap mesenchyme; PTA, pre-tubular aggregate; RV, renal vesicle. (C) Isolation of Six2^GFP^ NPC from Six2^GC^ mouse kidneys by FACS. (D–G) Single-cell RNA seq of E16.5 Six2^GFP^ NPC. (D) tSNE plot showing ten cell clusters; (E) Heatmap with the expression pattern of the top five cluster-specific genes in the ten clusters shown in D. (F) Violin plots showing the expression pattern of progenitor markers, *Cited1* and *Six2*, and the differentiation marker *Lhx1*. (G) Feature plot of *Six2* (marker of cap mesenchyme) and *Lhx1* (marker of differentiating NPC). Cells with high expression of *Six2* are in red and the cells with high expression of *Lhx1* are in blue. Cells with high expression of both *Six2* and *Lhx1* are in green. The FeaturePlot function in Seurat R that shows co-expression of these two genes was used to generate this plot. There is little if any overlap seen in expression pattern.

The nephrogenic niche consists of ureteric bud tip cells, surrounding crescent-shaped cap mesenchyme and adjacent stroma (Fig. 1A). The cap mesenchyme houses NPC, which are in turn composed of an early progenitor Cited1^+^/Six2^+^ compartment and a transit Cited1^−^/Six2^+^ compartment that subsequently differentiate into the pre-tubular aggregate, the precursor of the renal vesicle, the earliest epithelial precursor of the nephron (Fig. 1A). Fig. 1B depicts a kidney section from E16 Six2^GC^ mouse co-stained for Six2 and GFP; the Six2^GC^ transgene expresses GFP in the cap mesenchyme under the Six2 regulatory elements (Kobayashi et al., 2008). Due to the long half-life of GFP protein, low levels of GFP persist in the pre-tubular aggregate (Fig. 1B). Accordingly, when Six2^GFP^ cells are sorted out by FACS from developing kidneys of Six2^GC^ mice, they generally represent both the cap mesenchyme as well as early differentiating cells (Fig. 1C).

To assess the diversity of Six2^GFP^ NPC, we applied droplet-based single-cell RNA sequencing to 10,524 GFP^+^ cells isolated from E16 Six2^GC^ mice. Quality controls are shown in Fig. S1. These 10,524 single cells were classified into ten clusters using *t-*distributed stochastic neighborhood embedding (t-SNE; Fig. 1D). A heatmap with the expression of the five most differentially expressed genes in these clusters is shown in Fig. 1E. Cluster 0 contain Six2^GFP^ cells that express nephron progenitor markers (e.g. Crym and Cited1). Clusters 1–3 contain cells that express cell cycle control genes, DNA replication and centrosome duplication (e.g. Lig1, Dtl, Ccnb1, Cenpf, Cenpa). Cluster 4 contains cells expressing heterochromatin regulators such as Cbx3, PDCd4 and Kdm6b. Cluster 5–7 contain cells that are primed for epithelial differentiation expressing genes such as Wnt4, Id2, Igfbp5, Ccnd1, Lhx1 and Jag1. Clusters 8 and 9 contain Six2^GFP^ cells that co-express stromal markers. A previously published study using scRNA-seq showed that individual NPC exhibit stochastic expression of stroma markers (Brunskill et al., 2014). Violin and t-SNE plots clearly distinguish cluster 7 as containing the differentiation marker, Lhx1, whereas remaining clusters (and especially Clusters 0–3) are more representative of the progenitor cells (Fig. 1F,G). In general, our findings are consistent with those of recent scRNA-seq studies of human kidney development (Lindstrom et al., 2018; Menon et al., 2018; Wang et al., 2018) demonstrating that the NPC are composed of mesenchymal progenitors actively engaged in cell division while others are poised to differentiate.

### The accessible chromatin landscape during Six2^GFP^ NPC development

To assess the maturational changes in open chromatin during NPC development, we applied the assay for transposase-accessible chromatin by high-throughput sequencing (Omni-ATAC-seq) (Corces et al., 2017) to fresh FACS 50,000 Six2^GFP^ cells harvested from embryonic (E13, E16) and perinatal (P0, P2) kidneys of Six2^GC^ mice (Fig. 1C) (*n*=3–4 biological replicates per age group). By gating fluorescence intensity, we also FACS isolated and compared Six2^GFP(high)^ cells and differentiating Six2^GFP(low)^ cells from P0 Six2^Gc^ mice kidneys (Fig. S2). These latter groups of cells (*n*=3 per group) are designated heretofore as P0-H and P0-L, respectively.

**Fig. 2.**
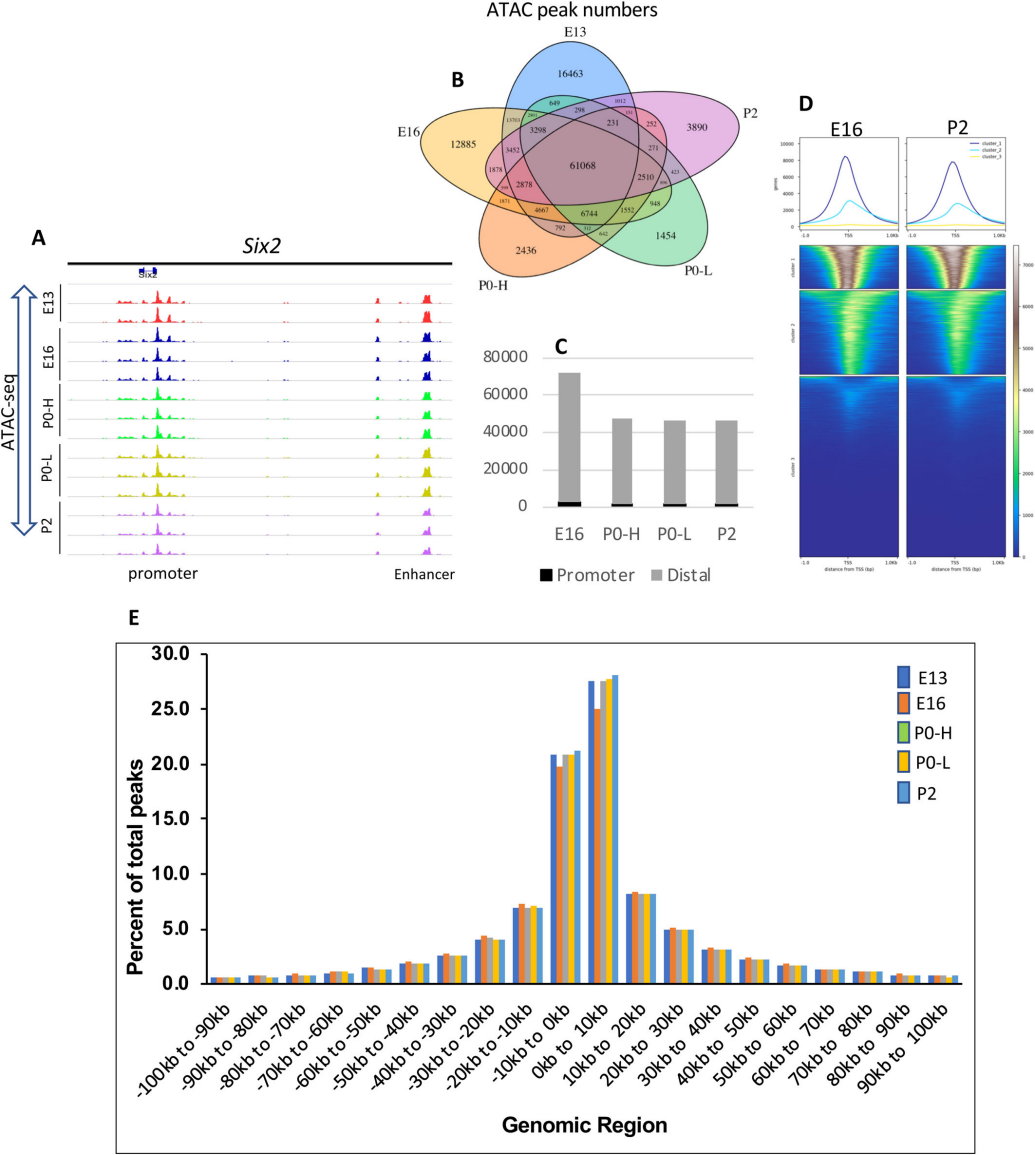
**Profiling of open (accessible) chromatin regions during NPC maturation by ATAC-seq.** (A) Representative ATAC-seq profiles of biological replicates from young (E13, E16) and old (P0, P2) NPC. P0-H and P0-L represent GFP^high^ and GFP^low^ perinatal NPC. (B) Venn diagram of ATAC-seq peaks per age group. (C) Number of ATAC peaks separated into promoter (±1 kb transcription start site) and distal genomic regions (−1 to −60 kb) in NPC of various ages. (D) Heatmap of ATAC peaks in the −1 to +1 kb around TSS in annotated genes at E16 and P2 NPC. (E) Distribution of ATAC peaks per genomic region in NPC of various ages.

ATAC-seq peaks, representing open (accessible) chromatin, were highly reproducible between biological replicates and showed a clear enrichment at the regulatory elements. As an example (Fig. 2A), in the *Six2* locus, the replicates show that the open chromatin regions (peaks) are concentrated in the promoter region and the annotated enhancer located 60 kb upstream of the TSS (Park et al., 2012). Analysis of ATAC-seq peaks revealed that young E13/E16 Six2^GFP^ NPC possess higher total number as well as distal open chromatin regions than old P0/P2 Six2^GFP^ NPC (Fig. 2B,C). Heatmap clustering of ATAC peaks in annotated genes around the TSS (−1 to +1 kb) did not reveal significant differences in open chromatin in E16 and P2 Six2^GFP^ NPC (Fig. 2D). Moreover, the genomic distribution (percent of total peaks per genomic region) was generally similar among the various age groups of Six2^GFP^ NPC (Fig. 2E).

### Six2^GFP^ NPC exhibit differential chromatin accessibility during development

**Fig. 3.**
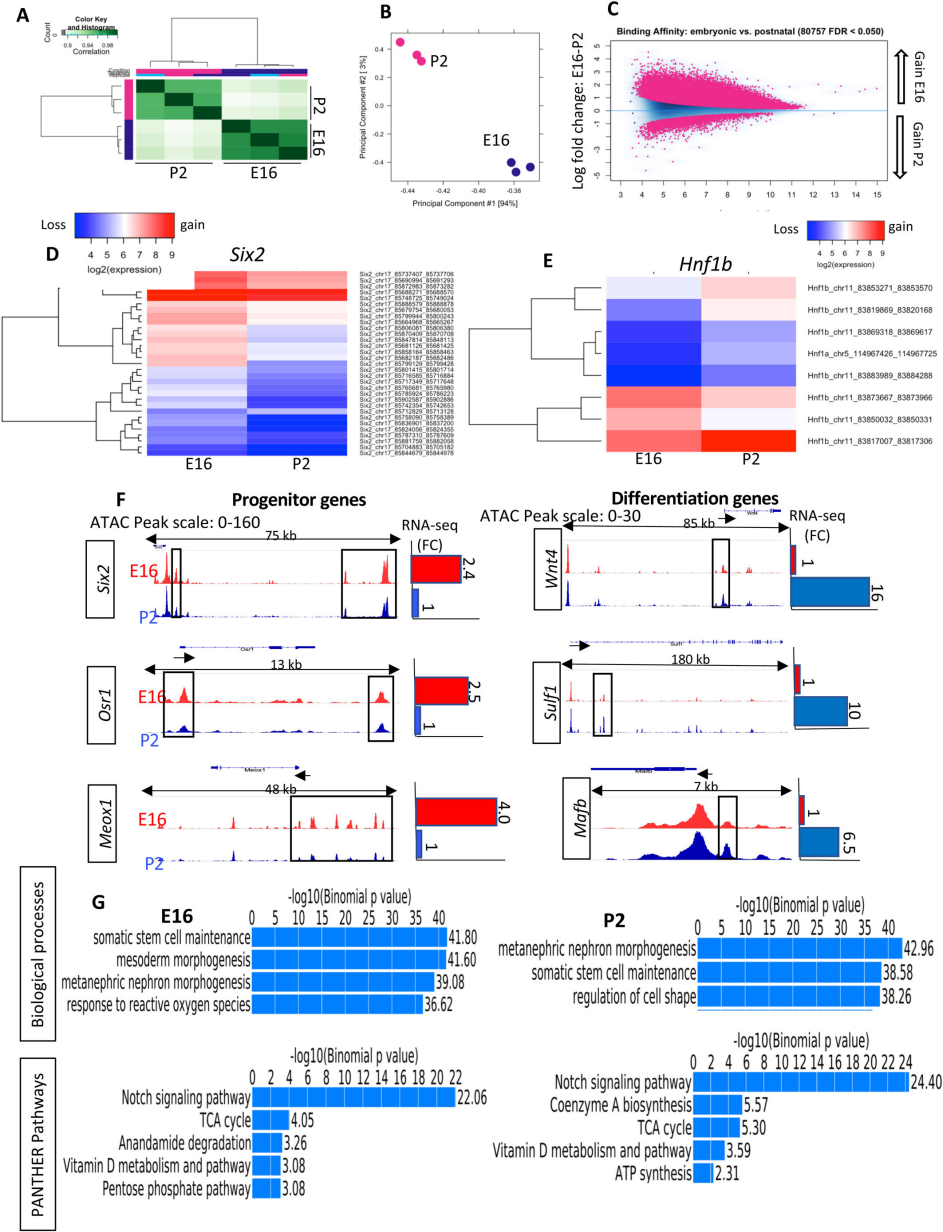
**Differential chromatin accessibility in embryonic (E16) and postnatal (P2) NPC.** Heatmap (A), Principal Component Analysis (B) and MA plot (C) of ATAC-seq peaks in E16 versus P2 NPC. (D, E) Heatmaps showing differential chromatin accessibility at the *Six2* and *HNF1b* loci at E16 versus P2 NPC. Red represents gain, whereas blue represents loss of open chromatin regions. (F) Open chromatin profiles correlate with expression of progenitor and differentiation genes during NPC maturation. The tracks represent ATAC-seq, whereas the bar graphs represent RNA-seq. RNA seq (fold change) were obtained from O'Brien et al. (2018). (G) GO functional annotation of ATAC peaks by GREAT analysis in E16 and P2 NPC.

We next compared the accessible (open) chromatin regions of E16 versus P2 as well as of P0-H versus P0-L Six2^GFP^ NPC using DiffBind R (http://bioconductor.org/packages/release/bioc/vignettes/DiffBind/inst/doc/DiffBind.pdf). The affinity analysis is a quantitative approach to assess for differential chromatin access at consensus peaks. This method takes read densities computed over consensus peak regions and provides a statistical estimate of the difference in read concentration between the two conditions. Fig. 3A and B show heatmap correlation plot and Principal Component Analysis, highlighting the differences in ATAC read concentrations between E16 and P2 experimental samples. The MA plot in Fig. 3C depicts the differences between ATAC peaks in the experimental samples by transforming the data onto M (log ratio) and A (mean average) scales, then plotting these values. Differential chromatin accessibility depicted in Fig. 3C is expressed as a log fold change of at least twofold and a *P*-value of <0.05 and reveals the relative gain of open chromatin regions in E16 NPC (above the 0 threshold line) as compared to the gain in P2 NPC (below the 0 threshold line). Differential chromatin accessibility at individual loci is illustrated in the heatmaps shown in Figs. 3D and E: NPC development is associated with a decline in open chromatin regions in the stemness factor, *Six2*, but a reciprocal increase in open chromatin in the pro-differentiation transcription factor *Hnf1-b* (Fig. 3D,E).

**Fig. 4.**
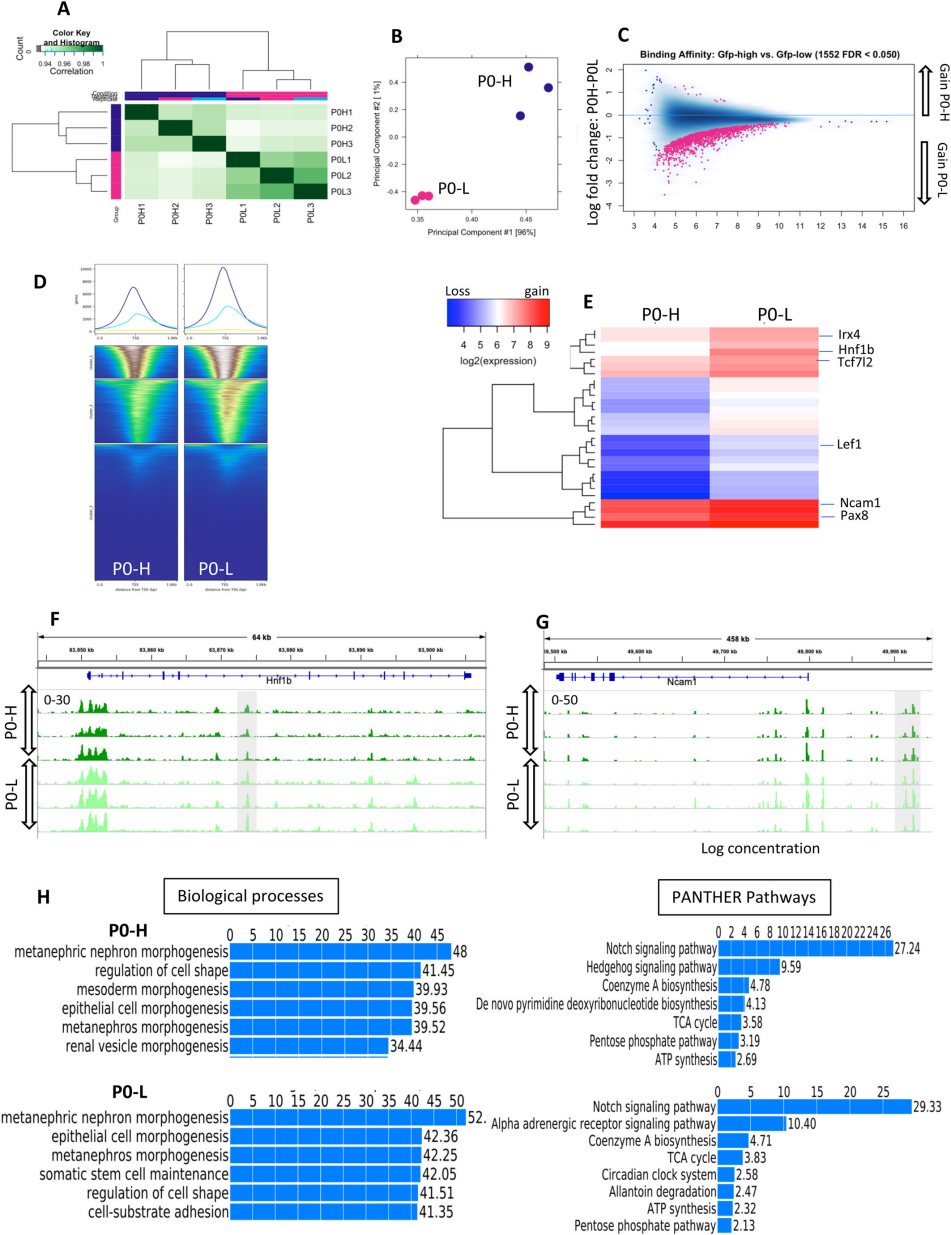
**Differential chromatin accessibility in progenitor Six2^GFP(high)^ (P0-H) and differentiating Six2^GFP(low)^ (P0-L) NPC.** Heatmap (A), Principal Component Analysis (B) and MA plot (C) of ATAC-seq peaks in P0-H versus P0-L NPC. (D) Heatmap of ATAC peaks in the −1 to +1 kb around TSS in annotated genes at P0-H and P0-L NPC. (E) Heatmap showing differential chromatin accessibility of selected differentiation genes in P0-H versus P0-L NPC. Red represents gain, whereas blue represents loss of open chromatin regions. (F,G) ATAC-seq tracks comparing P0-H and P0-L NPC in the HNF1b and NCam1 loci. Grey colored boxes highlight gain of open chromatin in P0-L versus P0-H. (H) GO functional annotation of ATAC peaks by GREAT analysis in P0-H and P0-L NPC.

We also compared the ATAC-seq signals representing open promoters and enhancers with published gene expression of NPC lineage (O'Brien et al., 2018). The results showed that progenitor genes in E16 Six2^GFP^ NPC (e.g. *Six2*, *Osr1* and *Meox1*) exhibit more open chromatin at distal elements and promoters than P2 Six2^GFP^ NPC (Fig. 3F). In comparison, poised/differentiation genes in P2 Six2^GFP^ NPC (e.g. *Wnt4*, *Sulf1* and *Mafb*) exhibit more open chromatin features than E16 Six2^GFP^ NPC (Fig. 4F). These findings indicate a good correlation between chromatin accessibility and gene expression levels during NPC maturation.

To understand the biological relevance of these dynamic changes in chromatin accessibility, we performed Gene Ontology analysis of the distal (−1 to −60 kb) regions in E16 and P2 Six2^GFP^ NPC. The results revealed common and distinct gene signature sets and pathways associated with their open chromatin regions (Fig. 3G). Among the common features are regulation of stem cell maintenance and nephrogenesis as well as Notch signaling and the TCA cycle. Distinct functional signatures include response to reactive oxygen species and pentose phosphate pathway (E16) and regulation of cell shape and ATP synthesis (P2).

### Chromatin accessibility in differentiating Six2^GFP^ NPC

In order to determine chromatin accessibility changes in differentiating Six2^GFP^ NPC, we compared ATAC peaks between P0-H and P0-L NPC, representing undifferentiated (GFP-high) and early differentiating (GFP-low) NPC. Fig. 4A–C shows a heatmap correlation plot, Principal Component Analysis and MA plot highlighting the differences in ATAC read concentrations and gain/loss of chromatin accessibility between P0-H and P0-L NPC. P0-L Six2^GFP^ cells also displayed increased accessibility around the TSS as compared to P0-H (Fig. 4D). Moreover, P0-L Six2^GFP^ cells gain chromatin accessibility in differentiation genes (e.g. *Lef1*, *Irx4*, *Hnf1b*, *Tcf7l2*, *Ncam1*, *Pax8*) (Fig. 4E). Figs 4F and G depict ATAC-seq tracks comparing two differentiation genes (*Hnf1b* and *Ncam1*) showing a differential increase in open chromatin (boxed regions) in P0-L NPC. Gene Ontology analysis of the distal (−1 to −60 kb) regions in Six2^GFP^ P0-H and P0-L revealed notable differences in categories related to regulation of cell shape and cell–substrate interaction pathways, albeit Notch signaling, the TCA cycle and ATP synthesis were prominent in both (Fig. 4H). Together, these findings illustrate a good correlation between accessible chromatin, representing regulatory regions and functional differentiation.

### Six2^GFP^ NPC maturation is associated with enhanced accessibility to the Bach2/AP1 transcription factors

We used ATAC-seq to identify transcription factor motifs within the accessible distal regions of maturing Six2^GFP^ NPC. HOMER revealed enrichment of the DNA-binding motifs of the core NPC transcription factors Six2 and Wt1 across all age groups reflecting their shared lineage (Table 1; Fig. S3). Hoxc9 and Tead DNA-binding motifs were also enriched among the top 21 most enriched motifs. Hoxa11 and Hoxd11 motifs, on the other hand, were enriched in younger E13 NPC. Notably, binding motifs for AP-1 transcription factor family, and BATF and Bach2 were progressively more enriched in older and differentiating Six2^GFP^ NPC (Table 1; Fig. S3).

**Table 1.**
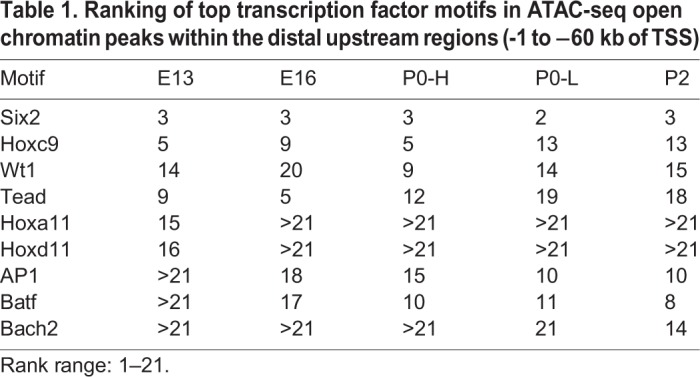
**Ranking of top transcription factor motifs in ATAC-seq open chromatin peaks within the distal upstream regions (-1 to −60 kb of TSS)**

### E13 and P0 Cited1^+^/Six2^+^ enriched NPC exhibit different epigenetic states

**Fig. 5.**
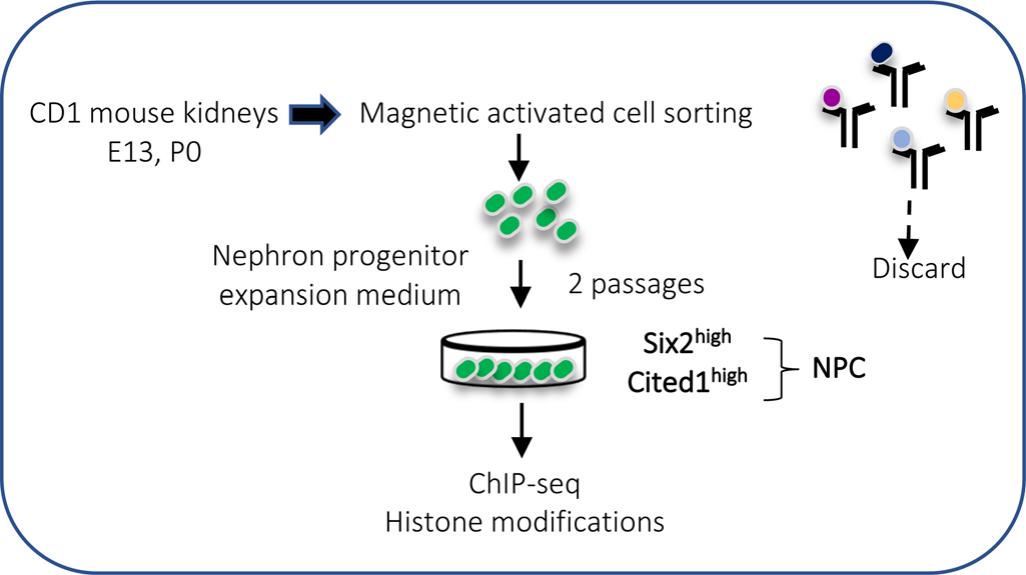
**Schematic of isolation of Cited1^+^-enriched NPC using Magnetic-Activated Cell Sorting (MACS).**

The results thus far have demonstrated that maturation of Six2^GFP^ NPC from E13 to P2 is accompanied by dynamic changes in the chromatin landscape. However, as shown in our single-cell RNA results, FACS isolated Six2^GFP^ cell are heterogenous and contain a small fraction of differentiating NPC. In addition, the niche microenvironment changes during maturation. Accordingly, in order to address whether NPC ‘age’ per se is a factor in the maturational changes of chromatin landscape, we applied magnetic-activated cell sorting (MACS) (Brown et al., 2013) to E13 and P0 kidneys from CD-1 mice (Fig. 5). E13 and P0 MACS-cells were then expanded in nephron progenitor expansion medium (NPEM) (Brown et al., 2013; Brown et al., 2015) for two passages to obtain near pure populations of Cited1^+^-enriched NPC. The MACS isolation, which is performed on wild-type tissue, also avoids the Six2^GFP^ transgene, in case it had a non-specific effect on the chromatin landscape.

**Fig. 6.**
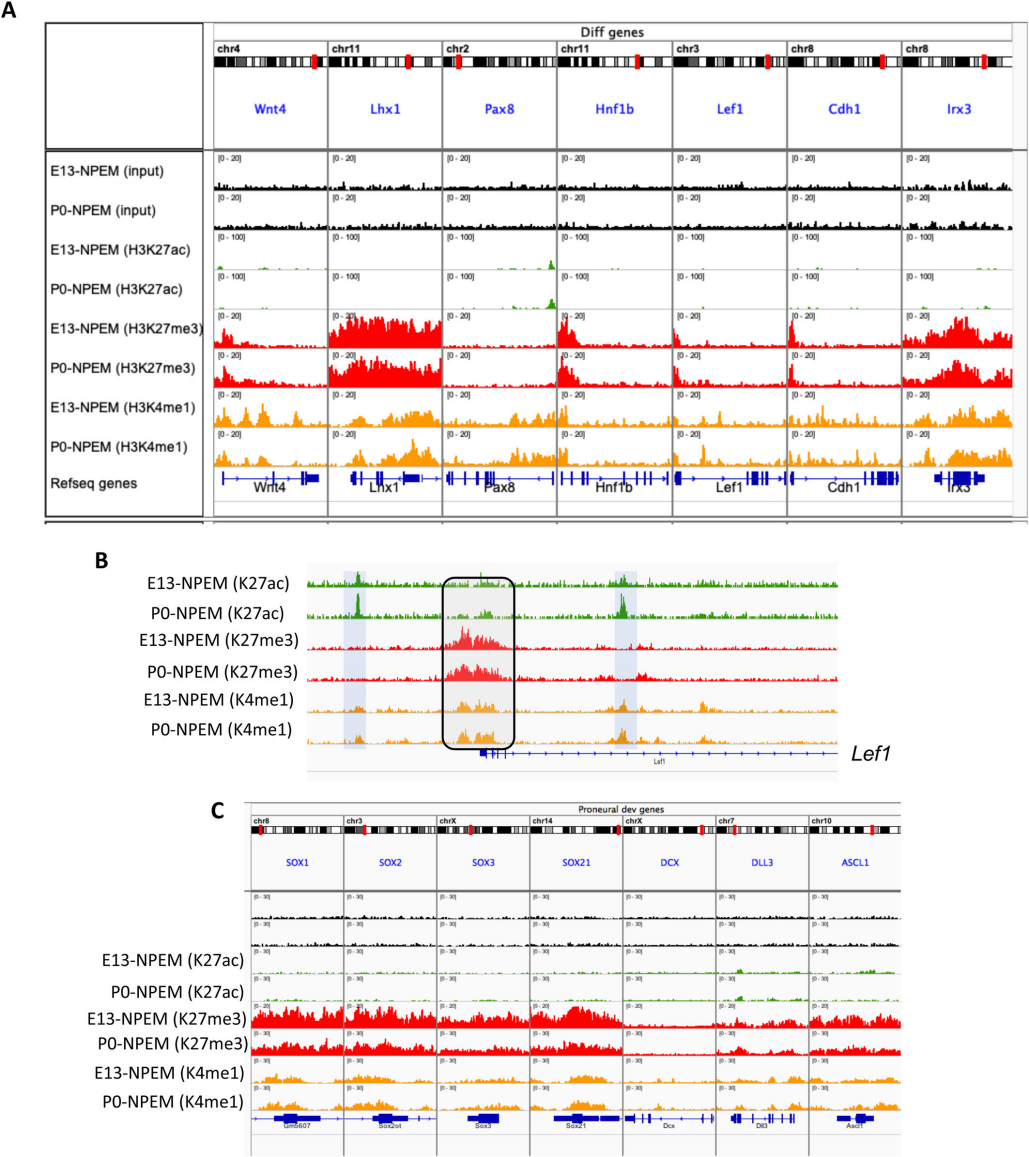
**ChIP-seq profiling of histone modifications in E13 and P0 NPC.** E13 and P0 NPC were isolated by Magnetic Activated Cell Sorting (MACS) then cultured in nephron progenitor expansion medium (NPEM) for two passages to enrich for Cited1^+^ NPC before they were subjected to ChIP-seq. (A) Poised differentiation genes are marked by bivalent H3K4me1/H3K27me3 marks. (B) ChIP-seq peaks representing putative active enhancers in the *Lef1* gene show differential gain in P0 versus E13 NPC (grey bars). The promoter region of the *Lef1* gene is occupied by bivalent H3K27me3/K4me1 peaks (grey box with black outline). In C, broad regions of repressive H3K27me3-marked chromatin cover the gene bodies of non-lineage genes.

ChIP-seq analysis was subsequently performed on NPEM-E13 and NPEM-P0 NPC to map active (H3K4me1/H3K27ac), repressed (H3K27me3) and poised (H3K4me1/H3K27me3) enhancers. The statistics of the ChIP-seq data are shown in Table S1. Fig. S4A–D depicts that pro-renewal pathway genes (Akt signaling, cell cycle, RTK signaling and epigenetic regulators) are all decorated with active histone marks, the exception being cell cycle inhibitors which are occupied by broad regions of the repressive mark H3K27me3 (Fig, S4B). In comparison, Fig. 6A shows that the genes required for mesenchyme-to-epithelium transition (e.g. *Wnt4*, *Pax8*) and downstream factors (e.g. *Lhx1*, *Lef1*) are occupied by bivalent chromatin regions (H3K4me1/K27me3), an epigenetic indication that these genes are poised for transcription. Fig. 6B illustrates an example of a poised differentiation gene, *Lef1*: note that the promoter region is bivalent (H3K27me3/H3K4me1) at E13 and P0; however, there is an age-related acquisition of two putative active enhancers (boxed regions). In comparison, non-lineage specific genes, such as pro-neural developmental genes, are occupied by broad domains of H3K27me3 which serve to restrain their expression (Fig. 6C).

**Fig. 7.**
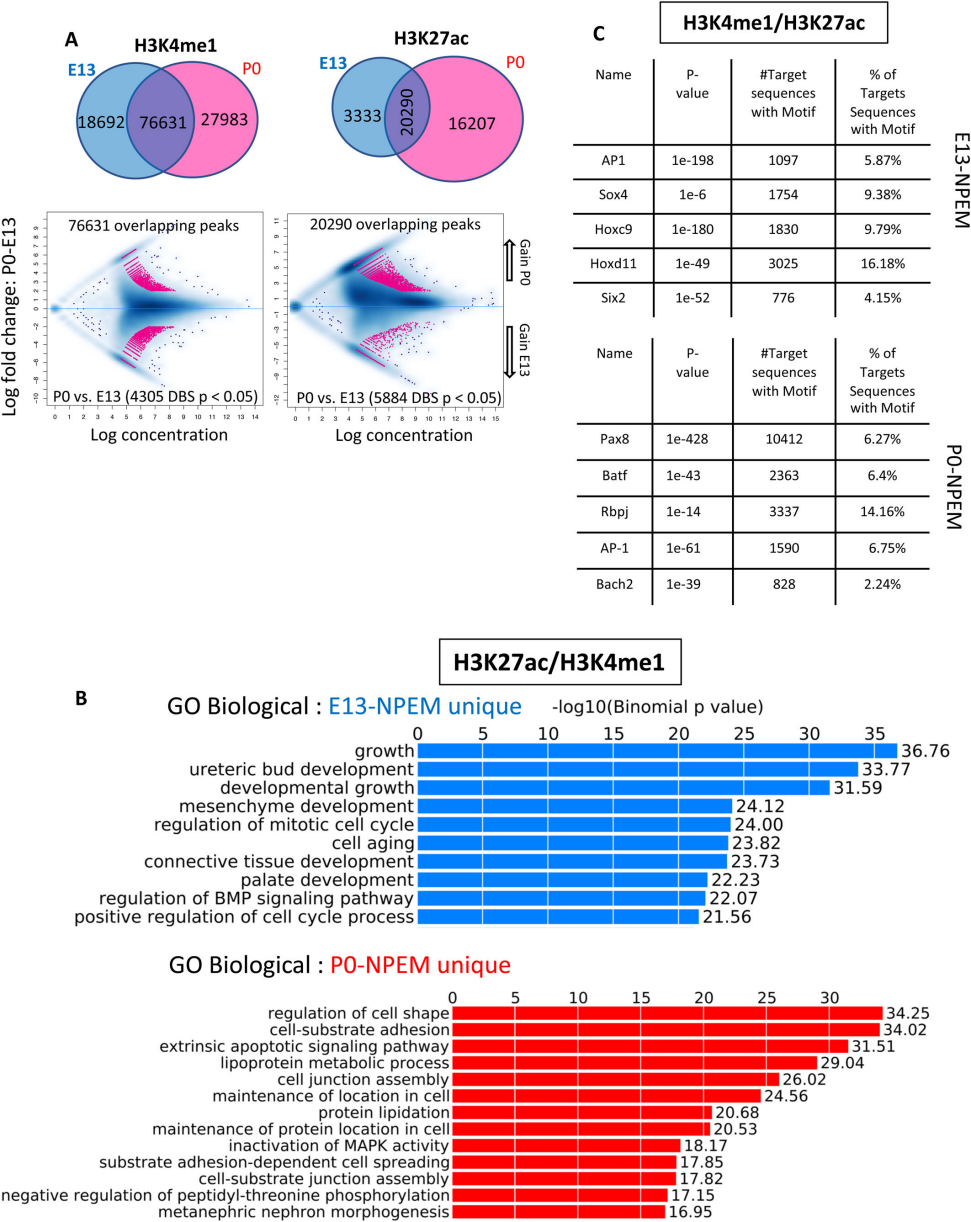
**ChIP-seq profiling of histone modifications in E13 and P0 NPC reveal active enhancer gain with age.** (A) (top) Venn diagrams of unique and shared H3K4me1 and H3K27ac active regions (top); (bottom) affinity binding analysis performed on the shared regions reveals a net gain of active H3K27-marked enhancers in P0 NPC. (B) GREAT analysis of the unique active enhancers in E13 versus P0 NPC reveals age-dependent biological processes. (C) HOMER analysis at E13 and P0 active enhancers defines distinct sets of enriched motifs in E13 and P0 NPC and reveals age-related gain of Bach2 and AP1 motifs.

Using ChIP-seq, we identified 18,692 primed enhancers (marked by H3K4me1) in E13-NPEM compared to 27,983 primed enhancers in P0-NPEM NPC (P0>E13+1.5-fold) (Fig. 7A). The numbers of H3K27ac-marked active enhancers were 3333 in E13-NPEM and 16,207 at P0-NPEM (P0>E13+4.8-fold) (Fig. 7A). Fig. S5 is a heatmap generated using DiffBind R showing the difference in H3K27ac/H3K4me1 concentration from E13 versus P0 NPC, illustrating the relative enrichment of this active histone mark in P0. GO interrogation of the differential H3K27Ac/H3K4me1-marked peaks in E13-NPEM and P0-NPEM NPC revealed a striking difference in biological functions: E13 NPC are more concerned with growth and cell cycle control, whereas P0 NPC were more engaged in differentiated functions such cell-cell and cell-junction assembly and inactivation of the MAPK pathway (Fig. 7B). Thus, during development, the transition from young to old NPC is associated with increased enhancer turnover reflecting the changes in NPC biology as they prepare to differentiate.

Motif enrichment analysis of E13-NPEM cells revealed that among the 15 most enriched active enhancers were the core factors AP1, Sox4, Hoxc9, Hoxd11 and Six2 (Fig. 7C). In P0-NPEM, the 15 most enriched active enhancers were the core TFs plus Pax8, Batf, Rbpj, AP1 and Bach2 (Fig. 7C). These results confirm the results observed using ATAC-seq (Fig. S3), implying a temporal association between age-related changes in chromatin accessibility and the binding of AP1/Bach2/Batf complexes. Our scRNA-seq indicates that Bach2 is expressed in Six2^GFP^ NPC along with Batf and the AP1 components Junb and Atf3 (Fig. S6A). Furthermore, interrogation of the GUDMAP/RBK database revealed that *Bach2* is expressed in the distal part of the renal vesicle, the earliest nephron precursor (Fig. S6B). Alignment of ATAC and ChIP-seq tracks also show the presence of two putative regulatory elements in the *Bach2* gene marked by the presence of consensus ATAC/H3K27ac/Six2/b-catenin peaks (Fig. S6C), suggesting that *Bach2* is a genomic target of canonical Wnt signaling, the principal driver of NPC differentiation.

### ATAC peaks correlate with NPC core transcription factor binding

**Fig. 8.**
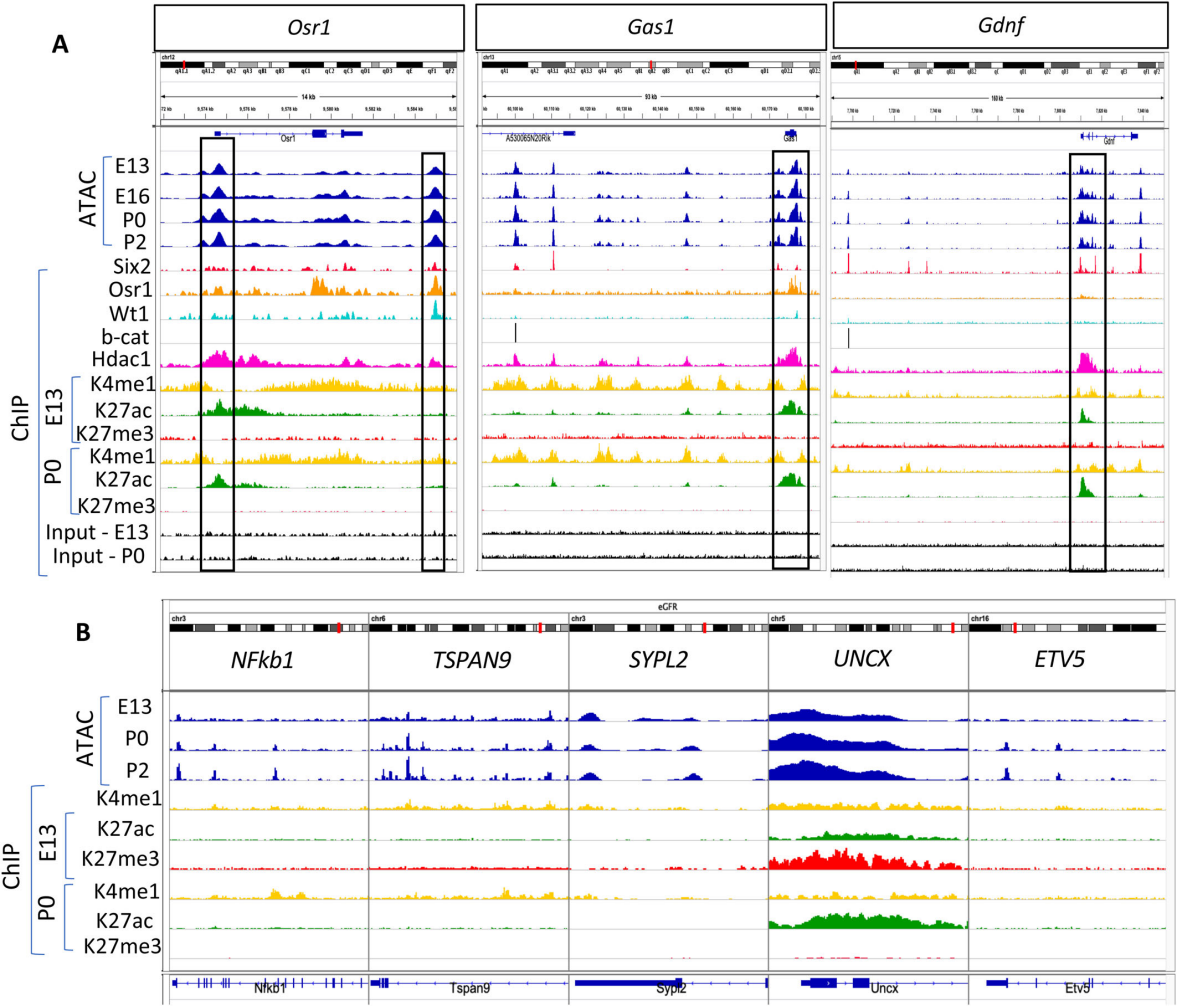
**Open chromatin profiles correlate with transcription factor binding at annotated and putative enhancers of NPC developmental regulators.** (A) IGV tracks showing integration of ATAC-seq and ChIP-seq peaks in promoters and enhancers of the progenitor genes, *Osr1*, *Gas1* and *Gdnf*. (B) ATAC/ChIP signature of genes found in genome-wide association studies of estimated glomerular filtration rate to demonstrate preferential mapping of associated variants to regulatory regions in kidney but not extra-renal tissues (Pattaro et al., 2016).

The open chromatin regions indicated by the ATAC peaks also correlated with the ChIP-seq peaks representing active enhancers (H3K4me1/H3K27ac), as well as with binding sites of the NPC core transcription factors Six2, Osr1 and Wt1 – previously shown by O'Brien et al. (O'Brien et al., 2018) (Fig. 8A). These findings also indicate a temporal coordination between chromatin accessibility at active enhancers and occupancy of the core transcription factor network of NPC. Of interest, we found that the binding of Hdac1 was a reliable indicator of open chromatin at the promoter region of actively transcribed genes where rapid cycles of histone acetylation and deacetylation are required for resetting the chromatin of active genes (Wang et al., 2009) (Fig. 8A).

### NPC chromatin profiles in genes implicated in renal function

We examined the ATAC signature of genes found in genome-wide association studies of estimated glomerular filtration rate and demonstrate preferential mapping of variants to regulatory regions in kidney but not extra-renal tissues (Pattaro et al., 2016). Although many of these genes were not active in NPC, they displayed open chromatin regions and were marked by H3K4me1 on putative primed enhancers (Fig. 8B). The homeobox transcription factor, *UNCX*, showed broad open chromatin regions that increase in width with NPC aging and is bivalent in E13 NPC but active in P0 NPC (Fig. 8B).

## DISCUSSION

Understanding the epigenetic mechanisms governing the nephron progenitor life span (Adli et al., 2015) is of great translational importance, since low nephron numbers predisposes an individual to hypertension and chronic kidney disease later in life. Importantly, monogenic mutations account for only about 20% of all cases of abnormal kidney development. Accordingly, there is a critical gap in our knowledge of how adverse prenatal environmental events affect renal development in the offspring. Moreover, a better understanding of the physiological, metabolic and epigenetic underpinnings of NPC aging may open new avenues to expand the nephron progenitor pool.

We previously interrogated the histone signatures of cultured metanephric mesenchyme cell lines (McLaughlin et al., 2013) and found that the onset of differentiation is accompanied by resolution of chromatin bivalency conforming to prior observations in pluripotent cells wherein genes essential for lineage commitment carry permissive histone modifications and appear poised for differentiation. In these studies, we observed that Wnt-responsive differentiation genes gain β-catenin/H3K4me3 binding and lose H3K27me3 at the TCF/LEF binding sites. By contrast, the promoters of progenitor genes show a gain of repressive marks and the loss of the active histone marks. Interestingly, immunolocalization studies in developing kidneys revealed that Six2+ nephron progenitors have higher levels of the repressive H3K9me2 and H3K27me3 marks and the histone modifiers, G9a, Ezh2 and HDACs (Liu et al., 2018; McLaughlin et al., 2014). While these studies were informative, additional work was needed to clarify whether the dynamic chromatin landscape observed in metanephric mesenchyme cell lines applies to native NPC.

In the present study, using native freshly isolated NPC across their lifespan, we demonstrate that as the NPC mature in the niche, chromatin accessibility to the regulatory regions of poised differentiation genes increases, likely preparing these gene networks for activation of the epithelial nephrogenesis program. These changes are not simply the results of changing proportions of the self-renewing and differentiating NPC populations but likely intrinsic, since young and old Cited1-enriched NPC grown in the same growth factor expansion medium also show significant differences in their chromatin enhancer landscape. We speculate that these maturational changes in chromatin accessibility are likely orchestrated by concomitant changes in the epigenetic machinery such as the Polycomb complex, ATP-dependent chromatin remodelers, DNA methylation and the NuRD/HDAC complex, that govern the access of master regulators to their cis-acting elements. Indeed, there is genetic evidence that perturbations of the epigenetic machinery disrupt the balance between NPC proliferation and differentiation *in vivo* (Denner and Rauchman, 2013; El-Dahr and Saifudeen, 2018; Liu et al., 2018; Zhang et al., 2018). A caveat in this study is that ChIP-seq was performed on E13 and P0 NPC cultured in NPEM for two passages. Previous studies have shown that stem and differentiated cells maintained in culture accumulate repressive chromatin marks and acquire progressively compact chromatin (Zhu et al., 2013). Therefore, future studies are required to examine and confirm our findings of the chromatin landscape in freshly isolated Cited1^+^ NPC.

A new finding of this study is that NPC maturation is associated with enhanced accessibility at the Bach2/Batf occupancy sites. Bach2 (BTB domain and CNC Homolog 2) is a transcription factor that is enriched in immune cells and plays a key role in regulation of the developmental B-cell transcriptional programs (Zhou et al., 2016). Previous studies have shown that Bach2 acts by recruiting Batf and competes with AP-1 for sequence-specific DNA binding on target genes (Kuwahara et al., 2016; Roychoudhuri et al., 2016). As mentioned earlier in the text, Bach2 was identified as part of the transcription factor signature of the distal renal vesicle, a compartment that receives high levels of Wnt9b/ß-catenin signaling (Brunskill et al., 2014). This leads us to the following hypothesis (Fig. S7): in young NPC, Six2/co-repressor complexes inhibit *Bach2* transcription. With further activation of Wnt/ß-catenin signaling and concomitant decline in Six2 levels, the Six2/TCF repressor is replaced by ß-catenin/TCF/co-activator complex leading to induction of *Bach2* transcription. The Bach2/Batf complex subsequently displaces the AP-1 complex inhibiting expression of AP1 targets (e.g. cell cycle genes). It is also conceivable that Bach2 targets differentiation genes to activate them. To our knowledge, studies of renal development have not been reported in Bach2- or Bach1/Bach2-deficient mice. Future investigations of *Bach2* function in nephrogenesis and delineation of Bach2-target genes will shed light on some of these gene regulatory networks. If indeed Bach2 links the proliferation and differentiation pathways in the NPC, targeting Bach2 may be a useful tool to manipulate the fate and lifespan of the NPC in renal regenerative medicine.

## MATERIALS AND METHODS

### Isolation of NPC

NPC were isolated from E13.5, E16.5, P0 and P2 CD1 mice or *Six2GFPCre* (Six2^GC^) mice by magnetic-activated cell sorting (MACS) (Brown et al., 2015) or fluorescent-activated cell sorting (FACS). For ATAC-seq, we used one litter (average of five to nine animals) per sample (*n*=3 to four samples per age group). For ChIP-seq, we used two litters per age group. Animal protocols utilized in this study were approved by and in strict adherence to guidelines established by the Tulane University Institutional Animal Care and Use Committee.

### ATAC-seq

For sample library preparation we followed the Omni-ATAC method outlined previously (Buenrostro et al., 2015; Corces et al., 2017). Briefly 50,000 nuclei from FACS-sorted cells were processed for Tn5 transposase-mediated tagmentation and adaptor incorporation at sites of accessible chromatin. This reaction was carried out using the Nextera DNA Library Prep kit (FC-121-1030, Illumina) at 37°C for 30 min. Following tagmentation the DNA fragments were purified using the Zymo DNA Clean and Concentrator Kit (D4014, ZYMO Research). Library amplification was performed using the Ad1 and any of Ad2.1 through Ad2.12 barcoded primers (Buenrostro et al., 2015). The quality of the purified DNA library was assessed on 6% TBE gels as well as on a Bioanalyzer (2100 Expert software, Agilent Technologies) using High Sensitivity DNA Chips (5067-4626; Agilent Technologies Inc.). The appropriate concentration of sample was determined using the Qubit Fluorometer (Molecular Probes). Four nM samples were pooled and run on a NextSeq 500/550 Hi Output Kit (20024907; Illumina, Inc. San Diego, CA) and the NextSeq 500 Illumina Sequencer to obtain paired end reads of 75 bp. Three to four independent biological replicates were sequenced per sample.

### Read processing and normalization of data

The paired-end reads for each sample run across four lanes of the flow cell (20022408; Illumina) were concatenated to obtain one forward and one reverse fastq.gz file each. The quality of the reads was assessed using FASTQC (v0.11.7). The paired end reads were aligned to the mouse reference genome mm10 using Bowtie 2. The properly aligned reads were filtered for mitochondrial reads (Sam tools) and cleared of duplicates (Picard-tools, v1.77). Only paired reads with high mapping quality (MAPQ >30) were included in the downstream analysis. The narrow peaks were called using MACS2 using the following parameters (effective genome size=1.87e+09; - -nomodel -p 0.001 - - no lambda; band width=300, d=200; *P*-value cut off=1.00e-03). Normalized bigwig files were generated using bedtools. Annotation and Known as well as *de novo* Motif discovery was achieved with Hypergeometric Optimization of Motif Enrichment (HOMER). Gene ontology analysis was performed using GREAT analysis 3.0. Heatmaps, density plots and differentially mapped regions were generated using DiffBind (Bioconductor).

### Single cell RNA-seq

We performed gene expression profiling of approximately 10,000 individual cells by using 10× Single Cell RNAseq technique provided by 10× Genomics. We first obtained a single cell suspension where cell viability was 88% or higher. These cells were applied for GEM generation and barcoding using the manufacturer's protocol. 10x™ GemCode™ Technology allows the partition of thousands of cells into nl-scale Gel Bead-In-Emulsions (GEMs) with application of ∼750,000 barcodes to separately index each cell's transcriptome. After GEM reaction mixture, full-length barcoded cDNA were generated and amplified by PCR to generate sufficient mass for library construction. Following enzymatic fragmentation, end-repair, A-tailing and adaptor ligation, indexed libraries were generated and sequenced. We used Cell Ranger version 2.1.1 (10× Genomics) to process raw sequencing data and Seurat suite version 2.2.1 for downstream analysis. Filtering was performed to remove multiplets and broken cells and uninteresting sources of variation were regressed out. Variable genes were determined by iterative selection based on the dispersion versus average expression of the gene. For clustering, principal-component analysis was performed for dimension reduction. Top 10 principal components (PCs) were selected by using a permutation-based test implemented in Seurat and passed to t-SNE for visualization of clusters.

### ChIP-seq

Chromatin was prepared from E13.5 and P0 NPC using the Diagenode iDeal ChIP-seq kit for Histones. Samples corresponding to 0.5 million cells were resuspended in 100 µl of shearing buffer iS1 and sheared during eight cycles of 30 s ‘ON’/30 s ‘OFF’ with the Bioruptor Pico combined with the Bioruptor Water cooler. The shearing efficiency was analyzed using an automated capillary electrophoresis system Fragment Analyzer (High sensitivity NGS fragment kit) after RNAse treatment, reversal of the crosslinking and purification of DNA. Based on optimization conditions, we used optimal antibody quantity resulting in higher enrichment and lower background (1 µg each of anti-H3K4me1, anti-H3K27me3 and anti-H3K27ac). The antibodies used were the following: H3K27ac (Diagenode antibody, C15410196, lot A1723-0041D), H3K4me1 (Diagenode antibody, C15410194, lot A1862D), H3K27me3 (Diagenode antibody, C15410195, lot A1811-001P). H3K4me3 (Diagenode antibody, C15410003, lot 5051-001P) was used as a ChIP Positive Control. After the IP, immunoprecipitated DNA was analyzed by qPCR to evaluate the specificity of the reaction. The primers pair, Six2 Prom (Fwd3 _ Rev 4) was used as positive control for the H3K27ac mark, Mouse Negative Control Primer Set1 (commercially available from Active Motif) was used as negative control region. Myogenic differentiation antigen 1 (MYOD1) and Mouse Negative Control Primer Set 3 were respectively used as positive and negative control regions for the H3K27me3 mark. Finally, Paired box 8 (Pax8int) and Mouse Negative Control Set 1 were respectively used as positive and negative controls for H3K4me1 mark. An IP with a control isotype (IgG 1 µg) was also performed. 500 pg of DNA was subsequently used for library preparation using the MicroPlex v2 protocol. The ChIP samples (eight samples in total) were processed together. A control library was processed in parallel of the samples using the same amount of a control Diagenode ChIP's DNA. According to the protocol, 12 cycles of amplification were performed to amplify the libraries. After amplification, 1 µl of each library was loaded on BioAnalyzer for quality and quantified using the Qubit ds DNA HS kit. Reference genomes were obtained from the UCSC genome browser. Sequencing was performed on an Illumina HiSeq 2500, running HiSeq Control Software 2.2.58. Quality control of sequencing reads was performed using FastQC. Reads were then aligned to the reference genome using BWA v. 0.7.5a. Samples were filtered for regions blacklisted by the ENCODE project. Subsequently samples were deduplicated using SAMtools v. 1.3.1. Alignment coordinates were converted to BED format using BEDTools v.2.17 and peak calling was performed using Sicer. Peaks sets generated with peak calling analysis were analyzed using DiffBind R/Bioconductor package.

## Supplementary Material

Supplementary information
