# Supplementary material for: Defining the dynamic chromatin landscape of mouse nephron progenitors
Source: Biol Open. 2019 May 7;8(5):bio042754. doi: 10.1242/bio.042754 (PMC6550063; doi:10.1242/bio.042754)
Supplement: Supplementary information [file biolopen-8-042754-s1.pdf]

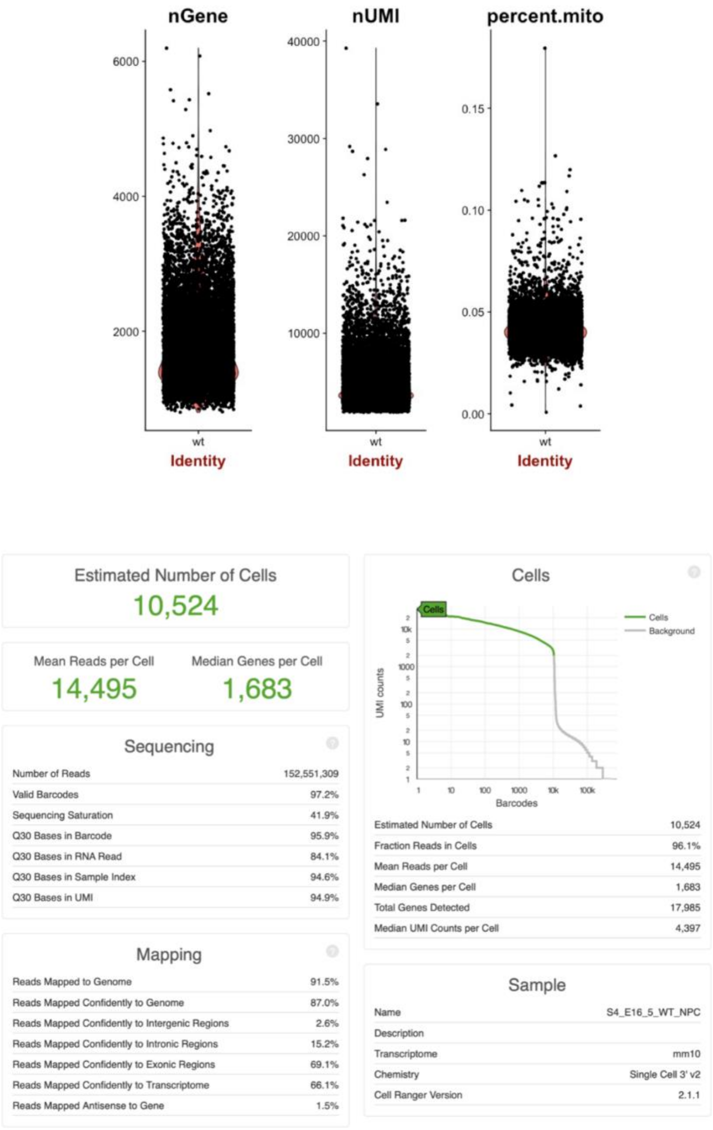

Figure S1. Quality controls for the single-cell RNA seq studies.

Biology Open • Supplementary information

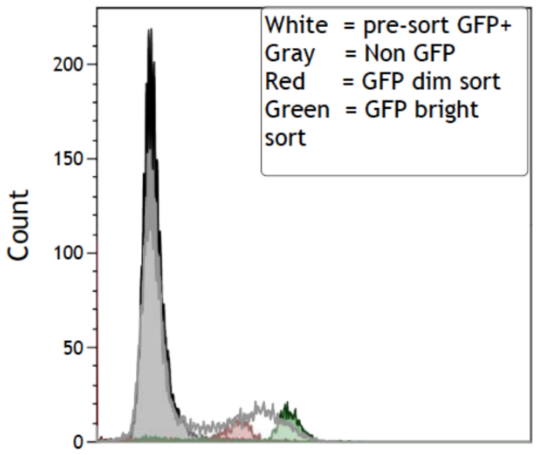

Figure S2. Fluorescence activated cell sorting of GFP(high) and GFP(low) NPC.

Biology Open • Supplementary information

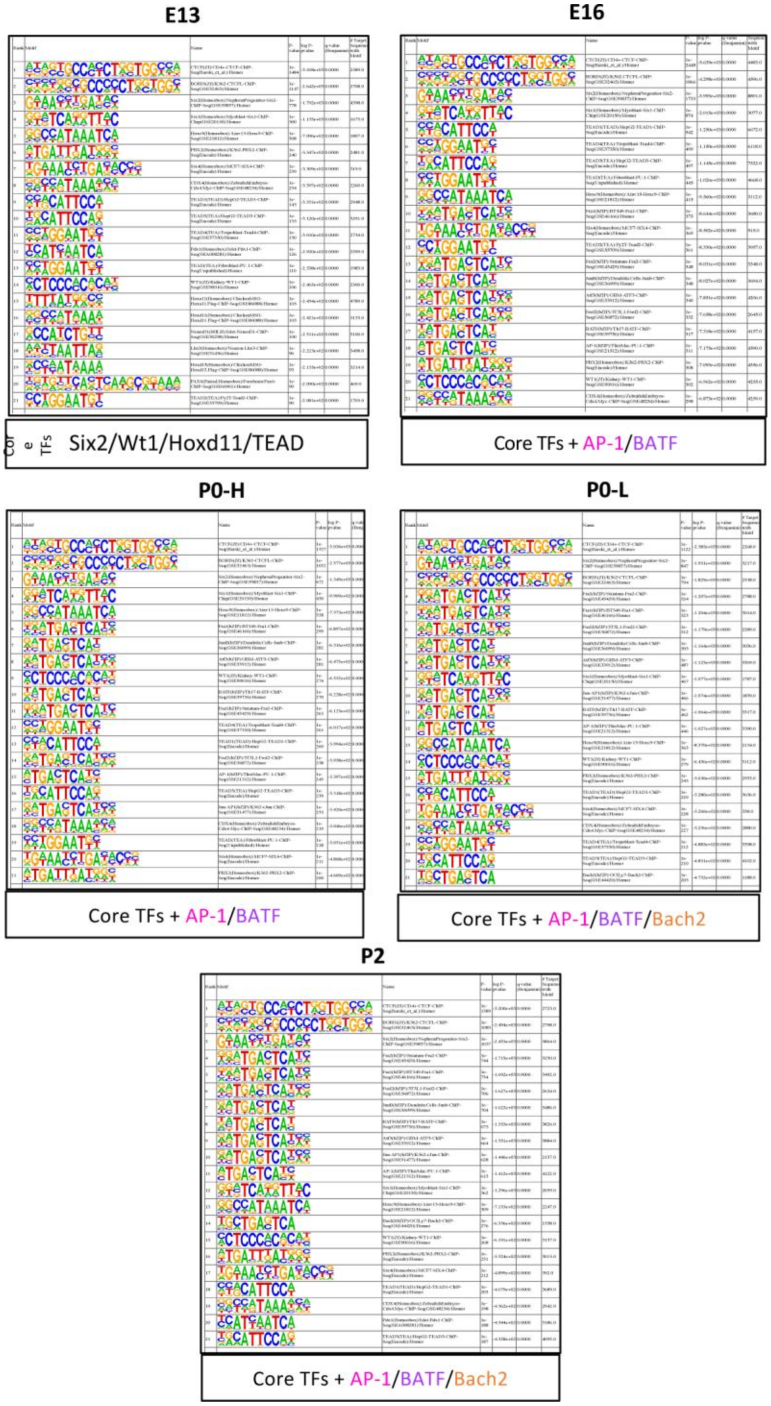

Figure S3. Motif enrichment analysis of ATAC-seq footprints identifies distinct sets of transcription factor binding sites in maturing NPC. Binding motifs for the core transcription factors (Six2/Wt1/Hoxd11/Tead) are enriched in NPC of all ages reflecting their shared lineage identity. Open chromatin regions of older NPC tend to be enriched in binding motifs for AP-1/Bach2/Batf.

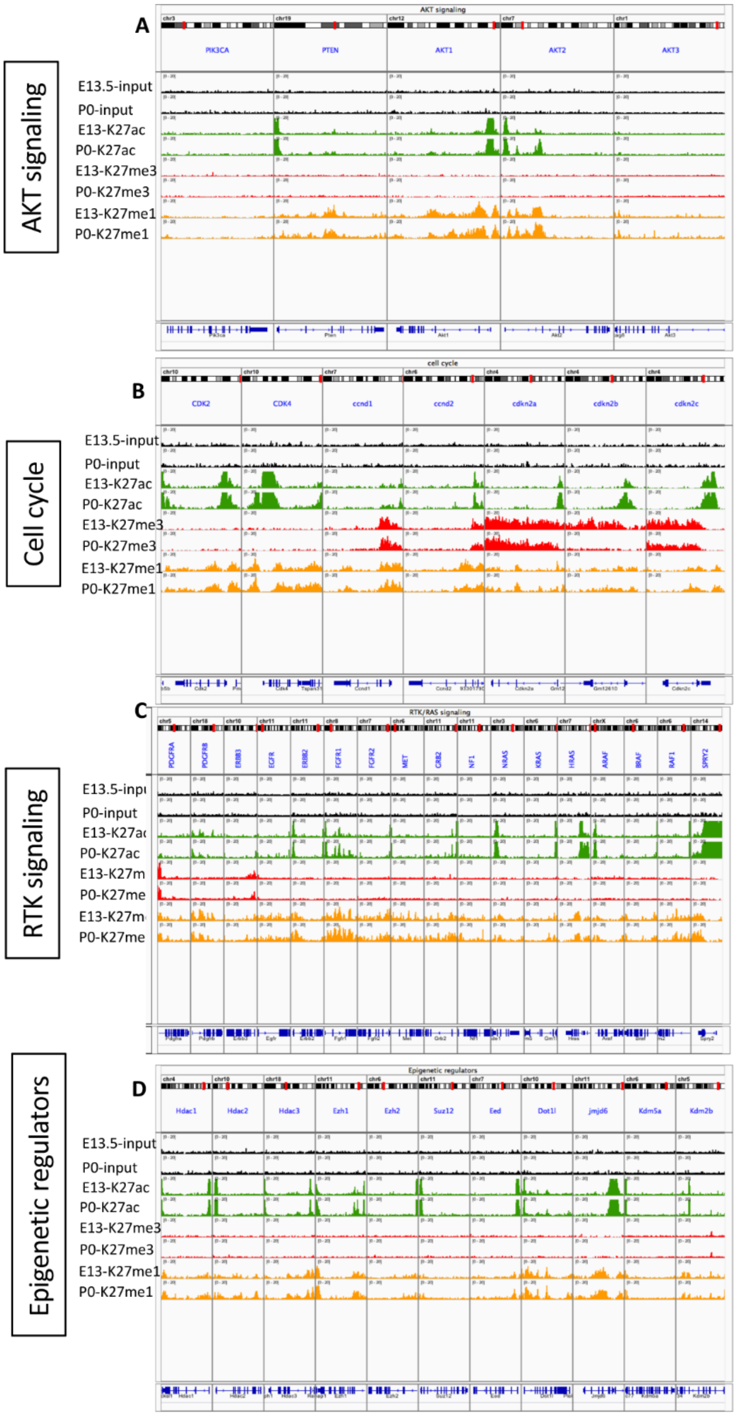

Figure S4. ChIP-seq profiling of histone modifications in representative active pro-renewal genes in young and old NPC.

Biology Open • Supplementary information

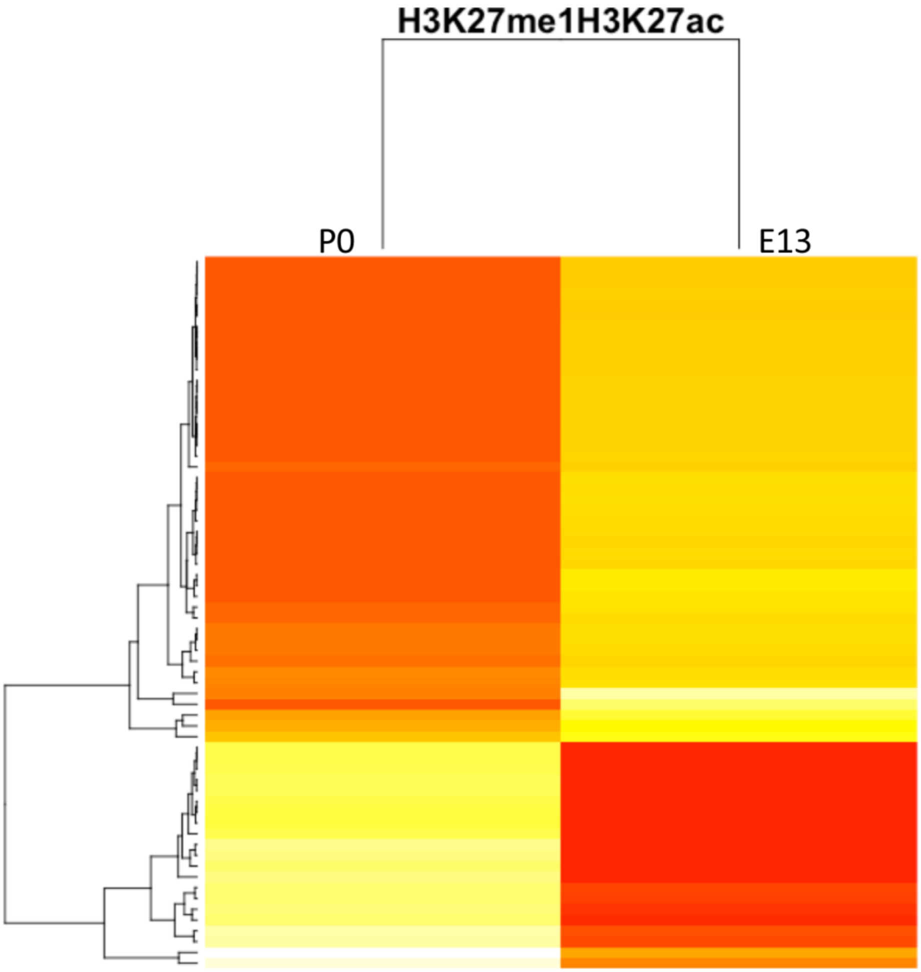

Figure S5. Heatmap of H3K4me1/H3K27ac active enhancer enrichment in E13 and P0 NPC.

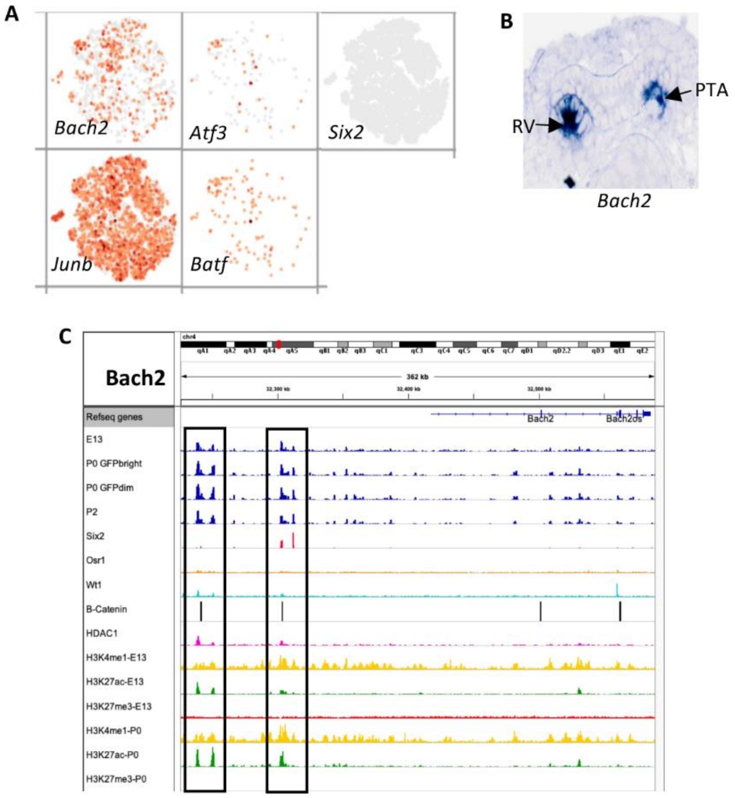

Figure S6. The transcription factor, *Bach2*, is expressed in the early differentiating nephron epithelium and displays active enhancers bound by Six2/β-catenin. (A) scRNA showing expression of *Bach2/Batf* transcripts and AP1 components in NPC. (B) *in situ* hybridization in TS23 stage developing mouse kidney showing that *Bach2* is expressed specifically in the pretubular aggregate (PTA) and distal renal vesicle (RV) (Source: Gudmap.org). (C) IGV tracks of the *Bach2* locus: note age-related opening of chromatin in the distal upstream region of *Bach2* (small arrow). These peaks are congruent with binding of ChIP-seq peaks for Six2 and β-catenin at two active enhancer elements (boxed regions).

Biology Open • Supplementary information

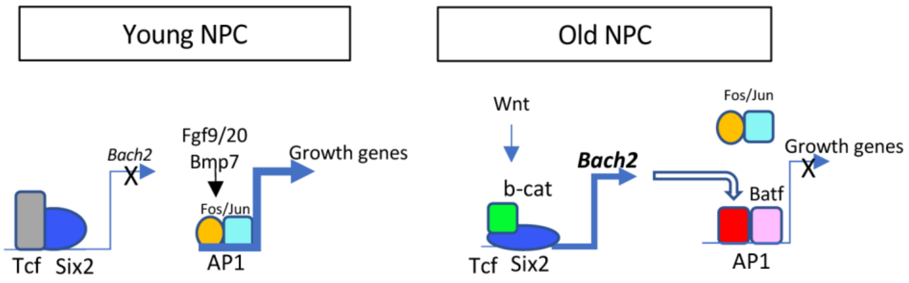

Figure S7. A hypothetical transcriptional model for chromatin state transitions during NPC maturation. In young NPC, Bach2 (a differentiation-promoting gene) is repressed by Six2/Tcf, whereas genes involved in stemness maintenance are activated by the growth factor/AP1 signaling module. During NPC maturation, Six2 levels decline and canonical Wnt activity is enhanced leading to Bach2 induction. Bach2/Batf in turn competes for DNA binding with the AP1 complex to turn off expression of renewal genes.

Biology Open • Supplementary information

Table S1: Statistics of ChIP-seq data.

Sample	Total reads	Uniquely aligned	Mapping efficiency	Peaks
E13.5 H3K4me1	54,619,120	39,237,983	0.7184	80,698
E13.5 H3K27me3	56,249,415	38,580,490	0.6859	5,949
E13.5 H3K27ac	64,813,226	38,273,136	0.5905	44,127
P0 H3K4me1	61,563,165	40,930,218	0.6640	77,947
P0 H3K27me3	65,761,659	39,721,356	0.6040	5,510
P0 H3K27ac	68,768,975	33,936,258	0.4935	27,442
